# Connective appendages in *Huberia bradeana* (Melastomataceae) affect pollen release during buzz pollination

**DOI:** 10.1111/plb.13244

**Published:** 2021-03-14

**Authors:** T. Bochorny, L. F. Bacci, A. S. Dellinger, F. A. Michelangeli, R. Goldenberg, V. L. G. Brito

**Affiliations:** ^1^ Programa de Pós‐Graduação em Biologia Vegetal Departamento de Biologia Vegetal Universidade Estadual de Campinas Campinas, São Paulo Brazil; ^2^ Department of Botany and Biodiversity Research University of Vienna Vienna Austria; ^3^ Institute of Systematic Botany The New York Botanical Garden Bronx NY USA; ^4^ Departamento de Botânica Universidade Federal do Paraná Curitiba, Paraná Brazil; ^5^ Instituto de Biologia Universidade Federal de Uberlândia Uberlândia Minas Gerais Brazil

**Keywords:** buzz pollination, carpenter bees, pollen release, sonication

## Abstract

- Floral structures, such as stamen appendages, play crucial roles in pollinator attraction, pollen release dynamics and, ultimately, the reproductive success of plants. The pollen‐rewarding, bee buzz‐pollinated flowers of *Melastomataceae* often bear conspicuous staminal appendages. Surprisingly, their functional role in the pollination process remains largely unclear. We use *Huberia bradeana* Bochorny & R. Goldenb. (*Melastomataceae*) with conspicuously elongated, twisted stamen appendages to investigate their functional role in the pollination process.
- We studied the effect of stamen appendages on pollinator behaviour and reproductive success by comparing manipulated flowers (appendages removed) with unmanipulated flowers. To assess bee pollinator behaviour, we measured three properties of buzzes (vibrations) produced by bees on *Huberia* flowers: frequency, duration and number of buzzes per flower visit. We measured male and female reproductive success by monitoring pollen release and deposition after single bee visits. Finally, we used artificial vibrations and laser vibrometry to assess how flower vibrational properties change with the removal of stamen appendages.
- Our results show that the absence of staminal appendages does not modify bee buzzing behaviour. Pollen release was higher in unmanipulated flowers, but stigmatic pollen loads differ only marginally between the two treatments. We also detected lower vibration amplitudes in intact flowers as compared to manipulated flowers in artificial vibration experiments.
- The presence of connective appendages are crucial in transmitting vibrations and assuring optimal pollen release. Therefore, we propose that the high diversity of colours, shapes and sizes of connective appendages in buzz‐pollinated flowers may have evolved by selection through male fitness.

## Graphical abstract

*Huberia bradeana* (Melastomataceae) has poricidal flowers with specialized stamen appendages that are crucial in transmitting vibrations and assuring optimal pollen release in buzz‐pollinated flowers.

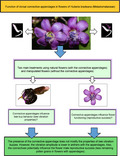

## INTRODUCTION

Strategies to ensure efficient pollen transfer include, for example, close morphological match between the flower and the pollinator (Buchmann 1983, Muchhala 2007), or restricted access to rewards, possibly requiring specialized pollinator behaviour, such as pollen extraction through vibrations (buzz pollination; Luo *et al.* 2008; Vallejo‐Marín *et al.* 2009, 2010; Amorim *et al.* 2017). The buzzing behaviour, *i.e*. the application of vibrations to flowers to remove pollen as food, is widespread across bees (65 families; Cardinal *et al.* 2018) and adaptations to buzz pollination have evolved in more than 72 plant families (ca. 22,000 species; Cardinal *et al.* 2018). Despite the widespread occurrence of buzz pollination in plants and bees, the underlying evolutionary drivers of this enigmatic interaction remain poorly understood (Vallejo‐Marín 2019).

Experiments have shown that pollen removal is controlled by essential bee vibrational properties, such as the frequency, duration and, mainly, amplitude of vibrations (De Luca & Vallejo‐Marín 2013; De Luca *et al.* 2013). Moreover, the optimal vibrational properties for maximum pollen release probably vary among plant species as a result of different floral structures, anther morphology and pollen properties (Buchmann 1983; Thorp 2000; Brito *et al*. 2020). Therefore, studying the structural and functional properties of poricidal anthers in relation to pollen release, as done in this study, has great potential to increase our understanding of the mechanisms underlying the evolution of buzz pollination.

Stamen connective appendages are common structures in many flowers that offer only pollen as a resource for bee pollinators (Cardinal *et al.* 2018). To date, the astonishing diversity of stamen appendage sizes, shapes, colours, intra‐staminal position and ontogenetic origin has primarily served systematic purposes (Renner 1993; Clausing & Renner 2001; Michelangeli *et al.* 2013). However, surprisingly little is known about their functional role in the pollination process (Buchmann 1983; Hermann & Palser 2000). Several functions have been proposed, such as that colourful or scented stamen appendages may increase floral attractiveness to pollinators (Luo *et al.* 2008; Velloso *et al.* 2018; Solís‐Montero *et al*. 2018), reward pollinators (Dellinger *et al.* 2014), serve as handles for buzzing bees when applying vibrations or help to position pollinators to optimize pollen transfer (Beattie 1971; Augspurger 1980; Renner 1989; Endress 1994; Han *et al.* 2008; Luo *et al.* 2008). An isolated study in passerine pollinated *Melastomataceae* showed that stamen appendages play a prominent role in pollen release *via* a bellows mechanism (Dellinger *et al.* 2014). Despite the prominence of stamen appendages in many buzz‐pollinated flowers, their possible and multiple functions have never been investigated experimentally.

**Fig. 1:**
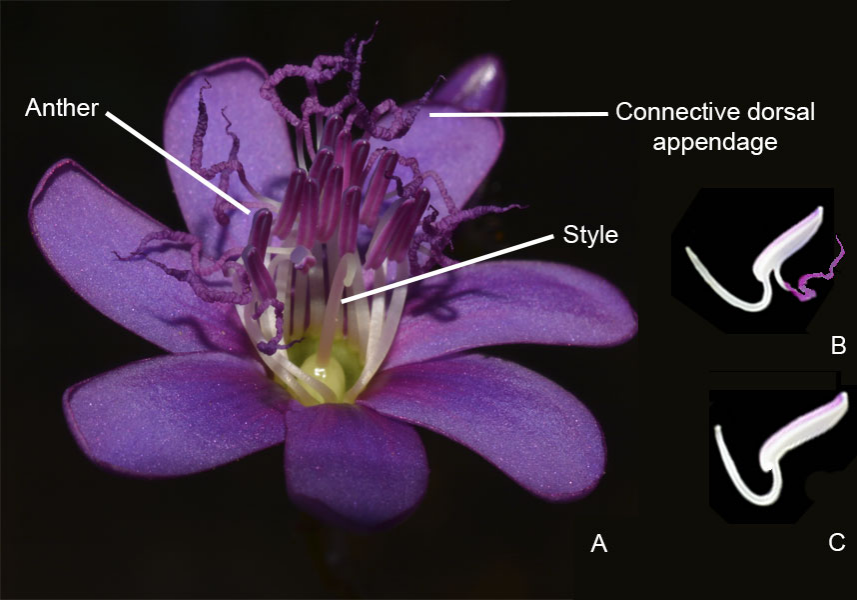
Flower of *Huberia bradeana* Bochorny & R. Goldenb. (Melastomataceae). (A) Anthers (stamens all grouped in a zygomorphic bundle that the bee grasps during buzz pollination), style and connective with dorsal appendages; (B) Natural stamen with connective appendage; (C) Manipulated stamen without connective appendage. (Photos: Renato Goldenberg and Thuane Bochorny).

Stamens of some *Melastomataceae* tribes (*e.g*. Melastomateae, Merianieae, Microliceae and Rhexieae) have evolved large connective appendages, which can be ventral and/or dorsal (Renner 1993; Clausing & Renner 2001; Michelangeli *et al.* 2013; Dellinger *et al.* 2019). Our study species, *Huberia bradeana* Bochorny & R. Goldenb. (formerly *Dolichoura spiritusanctensis* Brade), belongs to tribe Cambessedesieae (Bochorny *et al.* 2019) and presents stamens with a prominently prolonged, ‘whip‐like’ dorsal connective appendage (Fig. 1; Goldenberg & Tavares 2007). This long appendage was the reason why we selected this species as a model system to test the function of stamen connective appendages during the buzz pollination process (*i.e*. impact on bee vibrational properties and pollen release dynamics). Combining field observations, manipulative field experiments (artificial removal of appendages) and artificial buzzing experiments, we tested (i) whether the dorsal appendage of the stamen connective influences bee behaviour (vibrational properties) and (ii) whether appendages affect plant reproductive success (male and female fitness). Specifically, we addressed the following questions: (i) do the connective appendages influence the bee vibrational properties (frequency, duration and number of buzzes per visit); (ii) do the connective appendages affect the mechanical characteristics of flower vibration; and (iii) do the connective appendages potentially influence the reproductive success (pollen release from stamens and pollen deposition on stigmas)?

## MATERIAL AND METHODS

### Study species and site

*Huberia* is a genus of *Melastomataceae* comprising 37 species (Bochorny *et al.* 2019). *Huberia bradeana* is endemic to the Brazilian Atlantic Forest, growing in montane forests (between 600 and 800 m a.s.l.), usually close to water courses. It is a woody climber with perfect, nectarless, six‐ to seven‐merous flowers (Fig. 1). The petals are 5.0–7.9 × 2.4–3.7 mm in size and deep purple in colour at anthesis. The 14 stamens are isomorphic, grouped in a zygomorphic bundle, and the reflexed anthers are positioned opposite to the style at anthesis, with their ventral side turned towards the flower centre. All anthers have a purple, ‘whip‐like’, coiled dorsal connective appendage that is 2.0–2.5 times longer than the anther. The connective appendages are 4.1–6.2‐mm long and the anthers are about 2.1–2.5 mm in length. The anthers are also purple and slightly arcuate, with a dorsally inclined pore that directs the pollen away from the stigma upon release (Goldenberg & Tavares 2007). Flowers open in the morning and last only 1 day (Bochorny personal observation). The reproductive period is apparently very long: flowering specimens have been collected in August and from November through to March; fruiting specimens were collected between January and March (Bochorny personal observation). The fruits are capsules with long cuneate seeds (ca. 5 mm), dispersed by wind and/or gravity (Bochorny *et al.* 2019).

We conducted the experiments at Estação Biológica de Santa Lúcia (19°57′ S 40°32′ W) in the Municipality of Santa Teresa, Espírito Santo, Brazil. The reserve spans 440 ha along the banks of the Timbuí river, covered with montane Atlantic Forest from 500 to 950 m a.s.l., including some vegetation on rocky outcrops (Mendes & Padovan 2000). The climate in the region is of the Aw type, with well‐defined dry and rainy seasons (Mendes & Padovan 2000; Köppen 1918). Data for this study were collected during the month of January for three consecutive years from 2016 to 2018.

### Bee vibrational properties

**Fig. 2:**
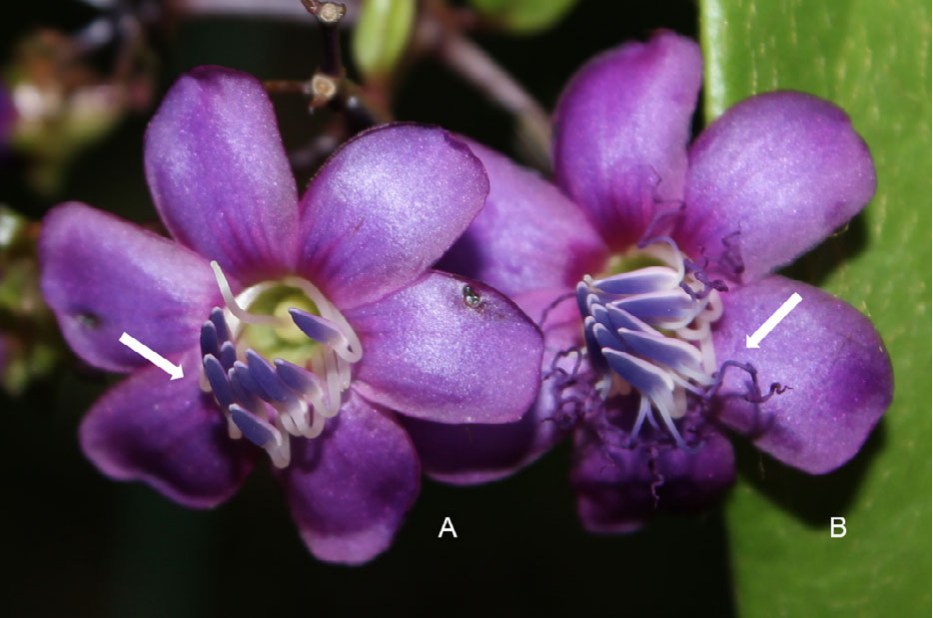
Flowers of *Huberia bradeana*. Two treatment groups: (A) manipulated flowers, in which connective appendages were artificially removed, and (B) natural flowers without manipulation (Photo: Thuane Bochorny).

**Fig. 3:**
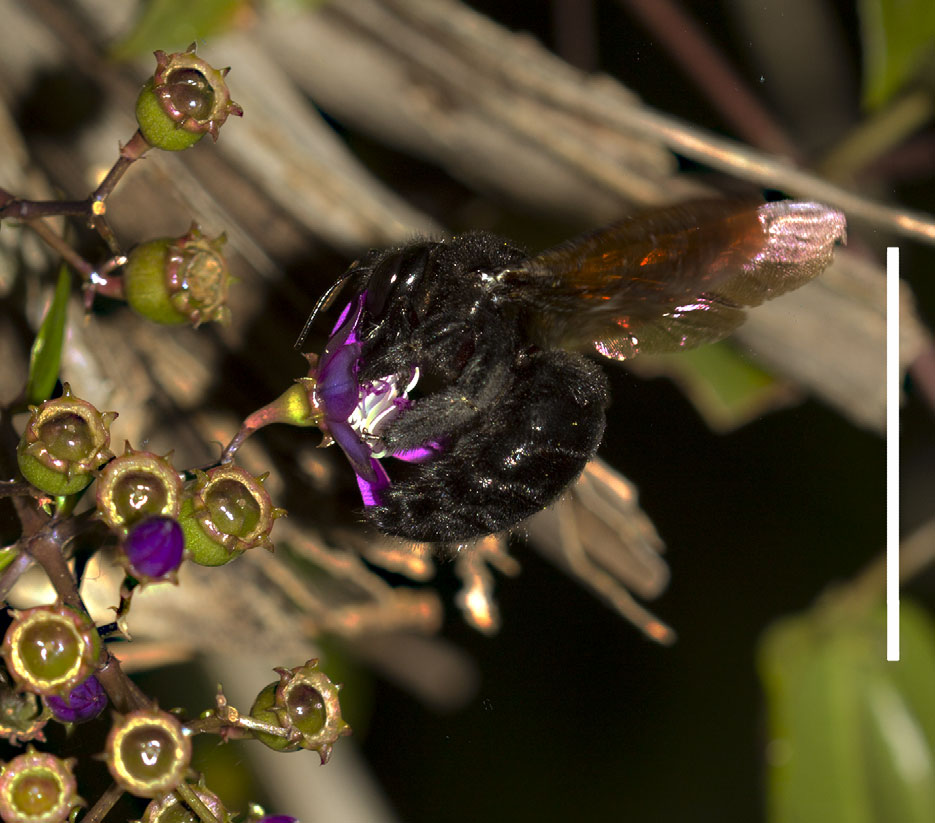
*Xylocopa* cf. *brasilionorum*. (Apidae) buzz pollinating *Huberia bradeana*. (Photo: Vinícius L. G. Brito).

To investigate whether the connective appendages influence bees’ buzzes, we prepared two treatment groups: natural flowers without manipulation and manipulated flowers in which the connective appendages were artificially removed (Fig. 2). We bagged flowers prior to their anthesis to prevent insect visitation. During predawn and before bee visits could occur, we removed the bags. We left half of these flowers unmanipulated (n = 52). In the other half (n = 56), we completely removed the connective appendages using tweezers. Manipulated flowers were colour‐coded with paper strips attached to the pedicel. When possible, we aimed to retain a balanced number of manipulated and unmanipulated flowers in the same inflorescence (Fig. 2). We then recorded the first visit of a legitimate bee pollinator to each flower. We considered a bee as a legitimate pollinator if it contacted the reproductive organs and if it performed buzz vibrations on the flower (Fig. 3).

During bee visits, we collected three natural vibration components: the buzz peak frequency (expressed in Hertz, Hz), the buzz duration (expressed in seconds, s) and the number of buzzes per flower visit. Such vibration characteristics were recorded for the carpenter bee species *Xylocopa* cf. *brasilianorum* (Linnaeus, 1767) and *Xylocopa* cf. *frontalis* (Olivier, 1789) during their visits to the flowers (Fig. 3). The large carpenter bees were previously recognized and differentiated in the field by the black abdomen in *X. brasilianorum* and red‐striped abdomen in *X*. *frontalis*. Thereafter, one specimen of each carpenter bee species was collected and identified in the lab by a bee specialist (Dr. Thiago Henrique Azevedo Tosta). Both bees were deposited in the Laboratório de Ecologia e Comportamento de Abelhas (LECA‐UFU) at the Federal University of Uberlândia. No other species of legitimate bee visitors were observed in *H. bradeana*.

We recorded bee vibrations while landing, while buzzing flowers and on taking off using a directional microphone (Directional Shotgun Csr Ht81; Yoga, China) connected to a digital recorder (ICD‐PX470; Sony, China) held at approximately 3 cm distance from the bee’s body. Since we were not able to adjust the microphone angle and position for all recordings, we did not consider the amplitude component in these experiments, according to the recommendations of De Luca *et al.* (2018). We analysed the temporal and spectral audio features of bee buzzes using the software Audacity (www.audacityteam.org).

### Flower vibration

To understand whether connective appendages affect the biomechanics of flower vibration, we conducted an experiment adapted from De Luca and Vallejo‐Marín (2013). We collected 40 freshly opened flowers and immediately transported them to the laboratory. In order that all flowers weighed approximately the same, thus reducing mass as a variable in the vibration experiment, we removed all pollen grains by vibrating the anthers with a hypodermic needle (medical syringe). Again, we created a set of manipulated (n = 20) and unmanipulated (n = 20) flowers. We attached each flower by its pedicel using entomological pins in a styrofoam plate covered by a black sheet of EVA material. We then attached a small piece (3 mm^2^) of adhesive tape at the base of the anthers in each flower. After preparing each flower, we lightly touched the base of the stamens of each flower with a handmade vibrational device (a metal rod coupled to a metal plate sound speaker connected to a computer; see Brito *et al*. 2020). We set the values of frequency = 238 Hz and amplitude = −35.6 dB and generated a pure tone sine wave of 5 s. These values of vibration components used in the playback system were tuned and calibrated to simulate the same bee vibrations collected as the mean value of 20 *Xylocopa* cf. *frontalis* specimens when visiting flowers in the field experiment. To record anther vibration, we positioned a laser Doppler vibrometer (PDV‐100 Portable Digital Vibrometer; Polytec) coupled to another computer 2.0 m from the focal flower and ensured that the adhesive tape and laser light beam were perpendicularly positioned in relation to each other. We adjusted the vibrometer velocity to 100, the lower pass filter to 22, while the high pass filter was not activated. Each flower was then vibrated for 5 s, and the anther vibration from the adhesive tape was measured. The input vibrations as well as the output flower vibration components (*i.e*. frequency and relative amplitude in decibels) were played and recorded using the software package Audacity (www.audacityteam.org).

### Pollen release by anthers and pollen deposited on stigmas

To assess pollen release, we used 25 flowers in each treatment (*i.e*. intact flowers or appendages removed). To estimate the amount of pollen released in each flower, we first removed one unvisited anther from each flower (control anther) and stored them in a micro‐centrifuge tube with 1 ml 70% ethanol. After the first pollinator visit, we immediately noted the carpenter bee species, then removed all other anthers from the same flower and stored them separately in 70% ethanol. We estimated the number of pollen grains in the control anther and in one anther from the visited flower from each flower using a haemocytometer slide under a microscope (100× objective lens) (Brito & Sazima 2012).

To estimate pollen deposition on stigmas, we used a total of 63 flowers (25 from unmanipulated flowers, 38 from manipulated flowers). Flowers that received a single visit by a pollinator were removed from the plant and all styles collected and mounted on microscope slides. Again, the visiting bee species was recorded. The number of pollen grains deposited on the stigmas was estimated by dividing the stigma in three sections of equal size. We estimated the amount of pollen covering the surface of the stigma in each section based on the following stigma load indices: 0 = 0%, 1 = 10%, 2 = 25%, 3 = 50% and 4 = 100% under a microscope (100× objective lens) (adapted from Brito & Sazima 2012). Note that our approach does not allow for controlling the amount of pollen initially present on a bee’s body and hence transferable to the stigmas. However, as this bias equally applies to both treatments, our comparisons are still robust.

### Data analyses

We used a linear mixed model to compare the peak frequency and buzz duration of bee sonication in flowers with and without connective appendages. In these analyses, the peak frequency and buzz duration were treated as response variables, while the flower treatment was the fixed factor; plant individual and bee species were considered random factors. After model fitting, the values of buzzing components in each flower treatment were compared using a type III ANOVA with Satterthwaite approximation for degrees of freedom (df). The same rationale was used to compare the number of bee buzzes per visit in each flower treatment, but in this case, we used a generalized linear mixed model with Poisson family distribution (link = log). In this model, the number of buzzes in each visit was the response variable, flower treatment was the fixed factor, flower identity and bee species were the random factors. We compared this model with a null model that considered only the random factors as explanatory variables using a Chi‐squared test.

To compare the vibrations recorded on anthers during artificial vibration in flowers with and without connective appendages, we also used a linear mixed model. As we did not find any difference between the frequencies of the harmonic peaks in either treatment, we compared only the relative amplitude on these peaks. In this case, the fitted model considered the recorded amplitude of anther vibrations as the response variable, and the treatment and harmonic peaks as fixed factors. The relative amplitudes were then compared using a type III ANOVA with Satterthwaite approximation for df. *Post‐hoc* tests were performed to compare the anther vibration amplitude between flowers with and without connective appendages in each harmonic peak using a pairwise *t*‐test with pooled SD and Bonferroni correction.

We adjusted a generalized linear mixed model using the Poisson family distribution (link = log) to compare the pollen grains remaining in the anther before and after the first pollinator visit in flowers with and without the appendage. In this model, the number of pollen grains in the anthers was considered as response variable, the time (before or after the first visit) and flower treatment were fixed factors, and flower identity as well as haemocytometer quadrant were considered random factors. A type III ANOVA with Satterthwaite approximation for df was used to compare the amount of pollen remaining in the anthers by each fixed factor after the model adjustment; pairwise *t*‐tests with pooled SD and Bonferroni correction were undertaken as *post‐hoc* tests. To test for differences in pollen receipt, we considered the index attributed in each section as the response variable and treatment as the fixed factor in a generalized linear mixed model with Poisson family; the stigma identity was considered as random factor. We repeated this analysis once, restarting from the fitted values of the first trial due to lack of convergence. We then tested the effect of treatment on the stigma pollen load index using the Wald Chi‐square test.

All statistical analyses and graphs were performed in the R environment (R Development Core Team 2012) using the following packages: lme4 (Bates *et al*. 2015), Rmisc (Hope 2013), plyr (Wickham 2011), ggplot2 (Wickham 2016), bbmle (Bolker 2017), lmerTest (Kuznetsova *et al.* 2017) and car (Fox & Weisberg 2019).

## RESULTS

**Fig. 4:**
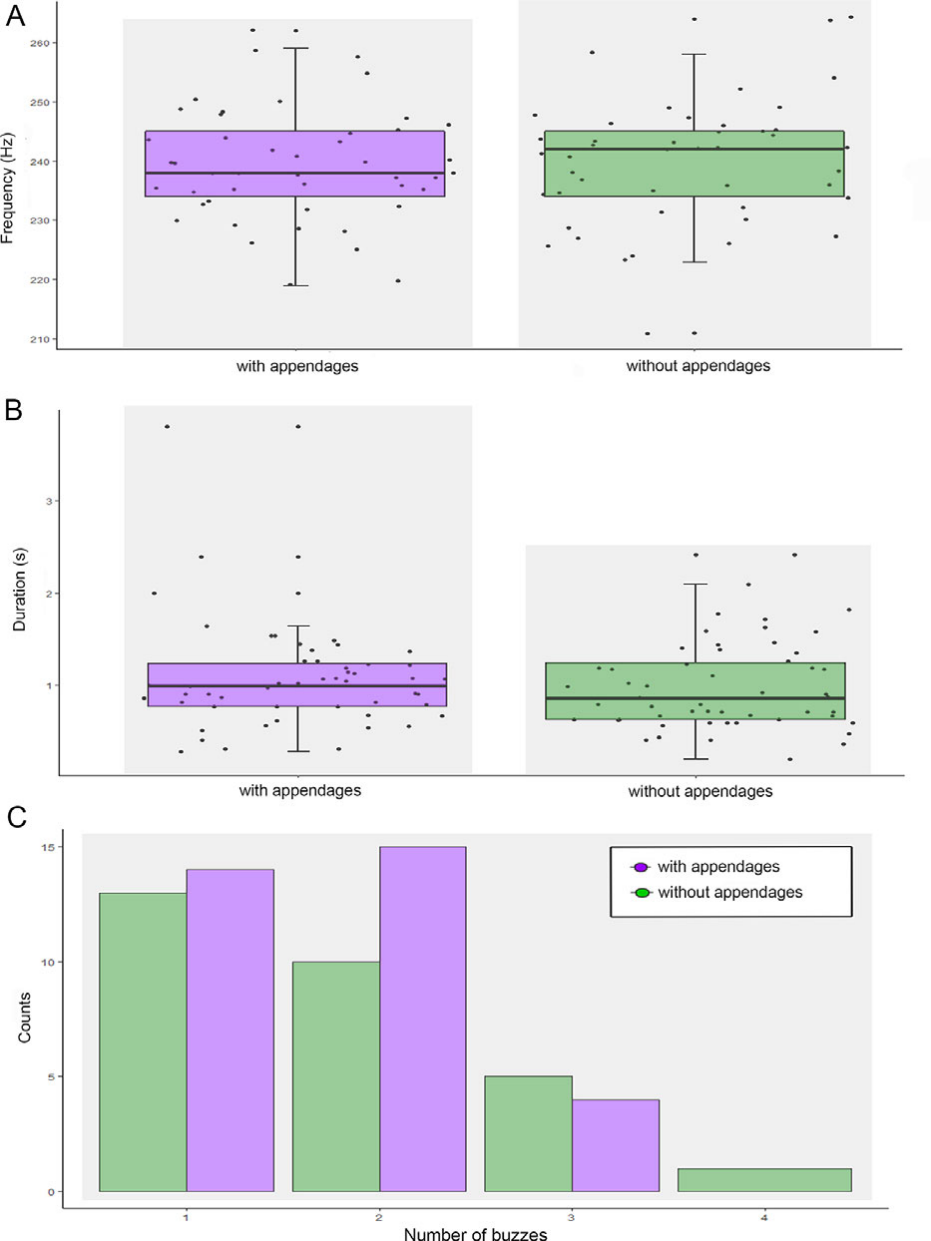
Comparison of bee sonication behaviour on *Huberia bradeana* flowers with or without connective dorsal appendages. (A) Frequency of vibrations; (B) Duration of vibrations; (C) Number of buzzes. Box plots show minimum and maximum values on the external whiskers and the first and third quartiles on the internal whiskers. Internal line represents the median.

**Fig. 5:**
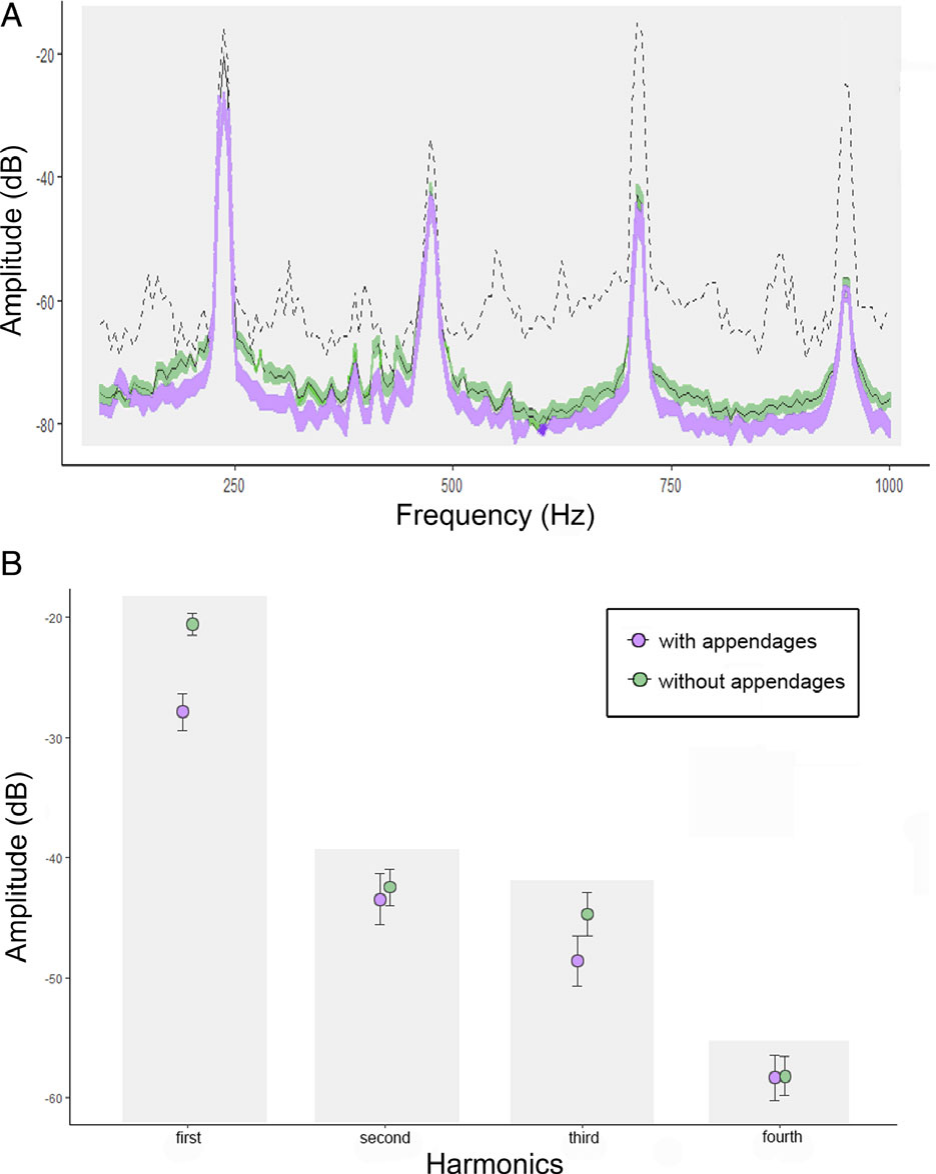
(A) spectrogram of flower vibration during artficial sonication in flowers of *Huberia bradeana* with (purple) and without (green) connective dorsal appendages (dashed line indicates vibration of the paper‐tag directly on the vibrator metal rod); (B) Comparison between relative amplitude of flower vibration in each harmonic peak during artificial vibration of flowers with and without appendages.

We recorded 108 bee vibrations on *H. bradeana* flowers, 96 being performed by *X*. cf. *brasilianorum* and 12 by *X*. cf. *frontalis.* The presence or absence of connective appendages did not influence the vibrational properties of these carpenter bees. Vibrational properties, such as peak frequency (*F* = 0.04, df = 1, *P* > 0.05), buzz duration (*F* = 2.52, df = 1, *P*> 0.05) and number of buzzes per visit (χ^2^ = 0.081, *P* > 0.05) did not differ between flowers with and without appendages (Fig. 4A–C, respectively). When using artificial buzzing experiments, however, the vibration relative amplitude of the first harmonic of anthers (Fig. 5A) differed between flowers with and without appendages (*F = *2.89, Df = 3, *P* < 0.05). In this harmonic peak, there was an amplitude reduction of 35% in anthers with appendages as compared to anthers without appendages (*P* < 0.01; Fig. 5B).

**Fig. 6:**
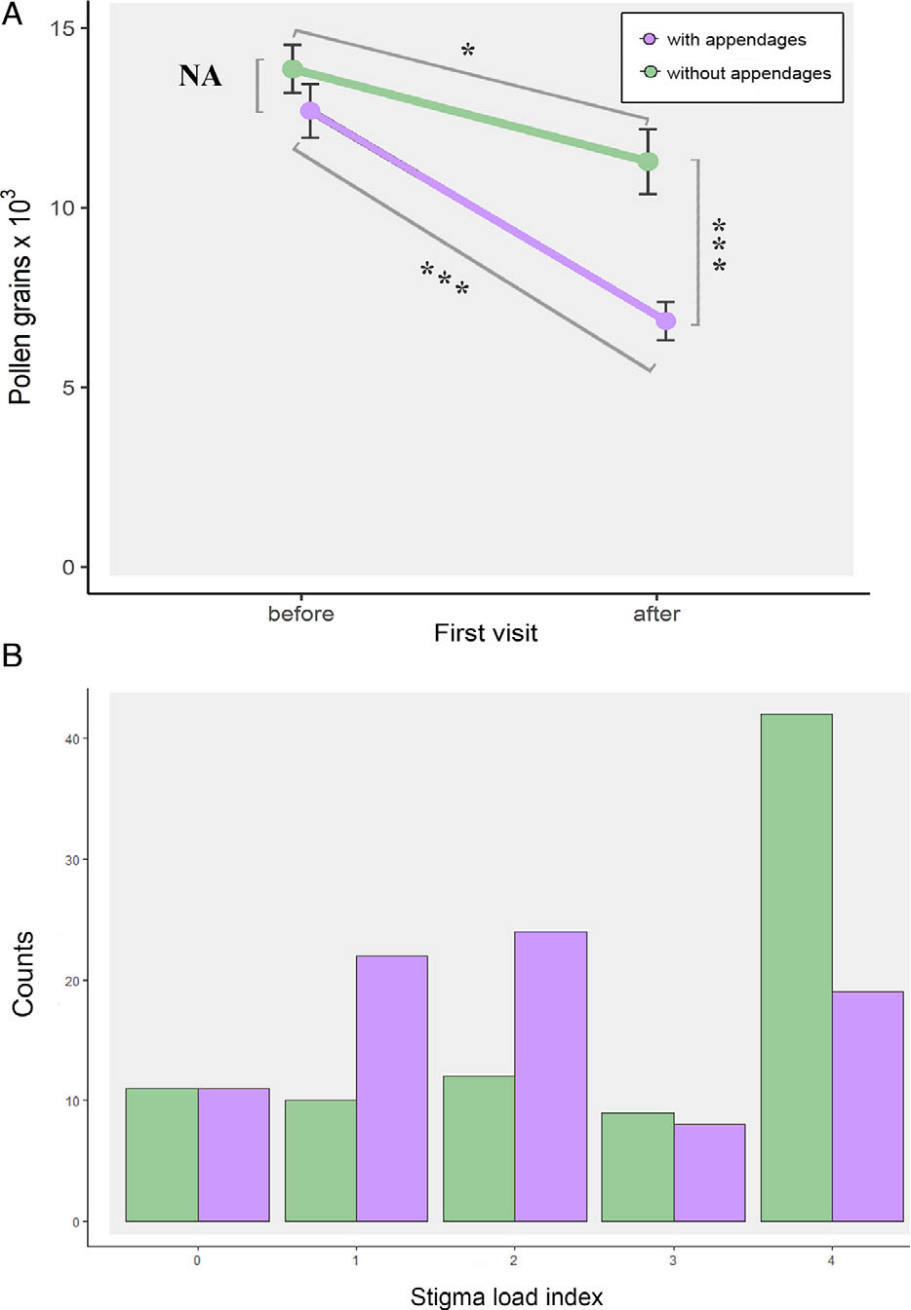
Effects of natural bee buzzes on (A) pollen released by anthers and (B) pollen receipt by stigmas of *Huberia bradeana* flowers with (purple) and without (green) appendages. (A) Number of pollen grains remaining in anthers before and after the first bee visit (pollen released) NA (not applicable); (B) Counts of stigma load indices after first bee visit (pollen receipt). The stigma load index was estimated by dividing the stigma in three sections. We used the following stigma load indices: 0 = 0%, 1 = 10%, 2 = 25%, 3 = 50% and 4 = 100% for section covered with pollen grains under a microscope (100× objective lens). *P < 0.05, ***P < 0.01.

*Huberia bradeana* flowers have 13.28 × 10^3^ ± 6.01 × 10^3^ pollen grains at the beginning of anthesis. After a single carpenter bee visit, we found 6.85 × 10^3^ ± 3.85 × 10^3^ pollen grains remaining in anthers of flowers with connective appendages, while 11.28 × 10^3^ ± 5.09 × 10^3^ pollen grains remained in anthers of flowers without the connective appendage. After the first visit, there were 39% fewer remaining pollen grains in flowers with appendages than in flowers without appendages (*P* < 0.01). We also found differences in the number of pollen grains before and after visits within treatments with (*P* < 0.01) and without (*P < *0.05) connective appendages. The interaction between time (before or after first visit) and flower treatment (with or without connective appendages) explained the number of pollen grains that remained in the anthers (*F = *9.8447, df = 1, *P* < 0.01; Fig. 6A). Stigmatic pollen load was marginally affected by the presence or absence of appendages (Wald χ^2^ = 3.4183, df = 1, *P* < 0.06; Fig. 6B).

## DISCUSSION

We found that the absence of connective appendages did not alter bee buzzing behaviour. However, the absence of appendages did change vibrational properties of the flower itself, since the relative amplitude of anthers lacking appendages was higher than that of anthers bearing appendages under artificial buzzing (simulating bee vibrational properties). Furthermore, connective appendages apparently also play a functional role in pollen release, since flowers without appendages released significantly less pollen than intact flowers. This suggests that complex connective appendages, as those of *Huberia*, may have evolved through selection, optimizing male fitness *via* altering flower mechanical properties (Minnaar *et al.* 2019). Female fitness does not seem to be directly affected by stamen appendages in *Huberia* as we found only a marginal effect of the presence or absence of connective appendages on stigmatic pollen loads.

### Bee vibrational properties

The connective appendages of *H. bradeana* do not modify the frequency and duration of bee vibrations. *Huberia bradeana* flowers are open for 1 day and apparently receive few visits per flower (Bochorny personal observation). It is reasonable to think that bees visiting *H. bradeana* try to optimize pollen collection by visiting as many flowers as possible and, in our experiment, absence of appendages seemed not to discourage the bees. These findings indicate that the connective appendages in *H. bradeana* probably do not function in attracting bees. Another indicator of appendages not functioning in pollinator attraction is the lack of a colour contrast between appendages and other floral parts, as it is common in other *Melastomataceae* species (Fig. 1; Velloso *et al.* 2018; Dellinger *et al.* 2019). We did not assess visitation rate and hence did not directly test for the attraction function of appendages. From the pollen deposition data, however, we know that appendages only marginally influence stigmatic pollen loads, so we also hypothesize that appendages do not function in the correct positioning of the pollinators. Taken together, the lack of evidence to support the attracting or positioning functions favours the idea that the appendages could function to enhance pollen release.

It is possible that the connective appendage also affects how strongly bees buzz the stamens (vibration amplitude), a component positively related to pollen release from poricidal anthers (Arroyo‐Correa *et al.* 2018; Rosi‐Denadai *et al.* 2018). In pollen flowers, the magnitude of the vibrations generated by the bee provides the energy that forcibly ejects pollen grains from the anthers (Buchmann & Hurley 1978), with higher amplitudes ejecting significantly more pollen grains (King & Buchmann 1996; De Luca *et al.* 2013b; Corbet & Huang 2014). Unfortunately, the acoustic set‐up used in this study to record bee sonication did not allow us to accurately compare potential differences in buzz amplitude between intact and manipulated flowers. Further controlled experiments are needed to accurately test whether bees adapt their buzzing amplitude to the presence or absence of connective appendages.

### Flower vibration and pollen release

In *H. bradeana*, stamen vibrational amplitude under artificial buzzing was lower in flowers with appendages, especially in the first harmonic. Despite there still being no clue to the effect of the secondary harmonics in pollen release from buzz‐pollinated flowers, flower traits, such as mass, stiffness, geometry and other material properties of anthers and associated floral structures are expected to affect the transmission of vibrations and ultimately pollen release (Michelsen *et al.* 1982; Vallejo‐Marín 2019; Brito *et al*. 2020). Moreover, it has already been discussed that the biomechanical structure of filaments can affect the transmission of vibrations by decreasing or increasing the amplitude produced by bees, potentially affecting pollen release (Buchmann & Hurley 1978; Harder & Barclay 1994; King & Buchmann 1996; Morgan *et al.* 2016; Switzer & Combes 2017; Brito *et al*. 2020). However, it is important to note that the laser vibrometer used in this study is capable of measuring the vibration in only one of the three flower vibration axes, while we know that flowers can vibrate differently in different spatial axes (Vallejo‐Marín 2019, Brito *et al*. 2020).

It is possible that stamens of *H. bradeana* bearing appendages transmit vibrations more thoroughly than stamens without appendages, resulting in higher pollen release in flowers with appendages, although this parameter has not been recorded by us. We hypothesize that the appendages optimize the kinetic energy diffusion throughout the connective tissue to the anther and then to the pollen grains (King & Buchmann 1996). This might be particularly important in plants with poricidal anthers that restrict pollen release through an apical slit or pore (Buchmann 1983). Because of this morphology, incidental contact between pollen and pollinator is not possible and removal of pollen from such anthers requires active vibration. In cases of high pollinator abundance, it is desirable to portion pollen strictly so that pollen can be dispersed to more pollinators, and thus reach more conspecific flowers (Harder & Thomson 1989). Conversely, releasing a large proportion of pollen in the first visit would be especially advantageous under scenarios of low bee–pollinator abundance, such as in the forest understorey (Sargent & Vamosi 2008).

Here, we have shown that the connective appendages of *H. bradeana* potentially enhance male fitness by releasing more pollen grains. In fact, in *Rhexia virginica* L., a pollen rewarding *Melastomataceae*, there is a negative relationship between the number of pollen grains released by poricidal anthers and pollen limitation. This finding suggests that higher pollen release potentially increases male success (Larson and Barrett 1999). Therefore, flowers with larger appendages may have an adaptive advantage directly linked to their reproduction *via* male fitness.

Our results indicate that the pollen loads deposited on the stigmas of flowers with and without appendages do not differ. Therefore, the connective appendages in *H. bradeana* appear to not be under selection by the female fitness component. However, the marginal *P*‐value and the apparent lower pollen load deposited on flowers with appendages (Fig. 6) indicates that more data could be helpful to shed light on this matter. Once confirmed that the connectives prevent high pollen load deposition on stigmas, it is possible that the connectives portion the pollen load in sequential bee visits. Since space on the stigma is limited, and if a flower has the chance of receiving more than one visit during its lifespan, this strategy will increase potential pollen donor diversity, potentially increasing female fitness.

In a phylogenetic perspective, *H. bradeana* belongs to the Neotropical *Melastomataceae* tribe Cambessedesieae, characterized by a large diversity in dorsal stamen connective appendages (Bochorny *et al.* 2019). Ancestors in the tribe lacked stamen connective appendages, and plants with simple or linear appendages evolved in *Huberia*. The majority of species have simple connective appendages (tiny and negligible tissue) or none at all (Bochorny *et al.* 2019). Due to the lack of sufficient empirical data, our understanding of the forces that drove the evolution of the complex appendage structures of *H. bradeana* is limited. More experimental work is needed to evaluate potential co‐evolutionary dynamics in plant–pollinator relationships that might explain the evolution of stamen connective appendages in *Melastomataceae*.

## CONCLUSIONS

Here we show that the conspicuous connective appendages of buzz‐pollinated flowers may not only function to attract bee pollinators, but also to govern pollen release dynamics. This functional interpretation of connective appendages implies that the evolution of anthers precipitates further changes in floral form. Since the presence of connective appendages directly affects pollen release dynamics, we propose that the high diversity of colours, shapes and sizes of connective appendages in buzz‐pollinated flowers may have evolved by selection through male fitness.

## References

[plb13244-bib-0001] Amorim T. , Marazzi B. , Soares A.A. , Forni‐Martins E.R. , Muiz C.R. , Westerkamp C. (2017) Ricochet pollination in *Senna* (Fabaceae) – petals deflect pollen jets and promote division of labour among flower structures. Plant Biology, 19, 951–962. 10.1111/plb.12607 28749609

[plb13244-bib-0002] Arroyo‐Correa B. , Beattie C.E. , Vallejo‐Marín M. (2018) Bee and floral traits affect the characteristics of the vibrations experienced by flowers during buzz‐pollination. Journal of Experimental Biology, 222(4), jeb198176. 10.1242/jeb.198176 30760551

[plb13244-bib-0003] Augspurger C.K. (1980) Mass flowering of a tropical shrub (*Hybanthus prunifolius*): influence on pollinator attraction and movement. Evolution, 34, 475–488. 28568699 10.1111/j.1558-5646.1980.tb04837.x

[plb13244-bib-0004] Bates D. , Maechler M. , Bolker B. , Walker S. (2015) lme4: Linear mixed‐effects models using Eigen and S4. R package version 1.1‐8. Available at: http://CRAN.R‐project.org/package=lme4 (accessed 02 May 2018).

[plb13244-bib-0005] Beattie A.T. (1971) Pollination mechanisms in *Viola* . New Phytologist, 70, 343–360.

[plb13244-bib-0006] Bochorny T. , Michelangeli F.A.M. , Almeda F. , Goldenberg R. (2019) Phylogenetics, morphology and circumscription of Cambessedesieae: a new Neotropical tribe of Melastomataceae. Botanical Journal of the Linnean Society, 190, 281–302. 10.1093/botlinnean/boz018

[plb13244-bib-0007] Bolker B. (2017) *bbmle*: Maximum likelihood estimation and analysis with the bbmle package. Available at: https://cran.r‐project.org/web/packages/bbmle/bbmle.pdf (accessed 02 May 2018).

[plb13244-bib-0008] Brito V.L.G. , Nunes C.E.P. , Resende C.R. , Montealegre‐Zapata F. , Vallejo‐Marín M. (2020) Biomechanical properties of a buzz‐pollinated flower. Royal Society Open Science, 7, 201010. 10.1098/rsos.201010 33047057 PMC7540744

[plb13244-bib-0009] Brito V.L.G. , Sazima M. (2012) *Tibouchina pulchra* (Melastomataceae): reproductive biology of a tree species at two sites of an elevational gradient in the Atlantic rainforest in Brazil. Plant Systematics and Evolution, 298, 1271–1279. 10.1007/s00606-012-0633-5

[plb13244-bib-0010] Buchmann S.L. (1983) Buzz pollination in angiosperms. In: Jones C.E. , Little R.J. (Eds), Handbook of Experimental Pollination. Van Nostrand Reinhold, New York, USA, pp 73–113.

[plb13244-bib-0011] Buchmann S. , Hurley J. (1978) A biophysical model for buzz pollination in angiosperms. Journal of Theoretical Biology, 72, 639–657. 10.1016/0022-5193(78)90277-1 672247

[plb13244-bib-0012] Cardinal S. , Buchmann S.L. , Russell A.L. (2018) The evolution of floral sonication, a pollen foraging behavior used by bees (Anthophila). Evolution, 72, 590–600. 10.1111/evo.13446 29392714 PMC5873439

[plb13244-bib-0013] Clausing G. , Renner S.S. (2001) Molecular phylogenetics of Melastomataceae and Memecylaceae: implications for character evolution. American Journal of Botany, 88, 486–498. 10.2307/2657114 11250827

[plb13244-bib-0014] Corbet A.S. , Huang S.Q. (2014) Buzz pollination in eight bumblebee‐pollinated *Pedicularis* species: does it involve vibration‐induced triboelectric charging of pollen grains? Annals of Botany, 114, 1665–1674. 10.1093/aob/mcu195 25274550 PMC4649695

[plb13244-bib-0016] De Luca P.A. , Bussière L.F. , Souto‐Vilaros D. , Goulson D. , Mason A.C. , Vallejo‐Marín M. (2013) Variability in bumblebee pollination buzzes affects the quantity of pollen released from flowers. Oecologia, 172, 805–816. 10.1007/s00442-012-2535-1 23188056

[plb13244-bib-0017] De Luca P.A. , Giebink N. , Mason A.C. , Papaj D. , Buchmann S.L. (2018) How well do acoustic recordings characterize properties of bee (*Anthophila*) floral sonication vibrations? Bioacoustics: The International Journal of Animal Sound and Its Recording, 29, 1–14. 10.1080/09524622.09522018.01511474

[plb13244-bib-0018] De Luca P.A. , Vallejo‐Marín M. (2013) What’s the “buzz” about? The ecology and evolutionary significance of buzz‐pollination. Current Opinion in Plant Biology, 16, 429–435. 10.1016/j.pbi.2013.05.002 23751734

[plb13244-bib-0019] Dellinger A.S. , Chartier M. , Fernández‐Fernández D. , Penneys D. , Alvear M. , Almeda F. , Michelangeli F. , Staedler Y. , Armbruster W.S. , Schonenberger J. (2019) Beyond buzz‐pollination – departures from an adaptive plateau lead to new pollination syndromes. New Phytologist, 221, 1136–1149. 10.1111/nph.15468 PMC6492237 30368819

[plb13244-bib-0020] Dellinger A.S. , Penneys D.S. , Staedler Y.M. , Fragner L. , Weckwerth W. , Schonenberger J. (2014) A specialized bird pollination system with a bellows mechanism for pollen transfer and staminal food body rewards. Current Biology, 24, 1615–1619. 10.1016/j.cub.2014.05.056 24998529

[plb13244-bib-0022] Endress P.K. (1994) Diversity and evolutionary biology of tropical flowers. Cambridge University Press, Cambridge, UK, pp 511.

[plb13244-bib-0023] Fox J. , Weisberg S. (2019) An R Companion to Applied Regression, 3rd edn. Sage, Thousand Oaks CA, USA. Available at: https://socialsciences.mcmaster.ca/jfox/Books/Companion/ (accessed 02 May 2018).

[plb13244-bib-0024] Goldenberg R. , Tavares R.A.M. (2007) A new species of *Dolichoura* (Melastomataceae) and broadened circumscription of the genus. Brittonia, 59, 226–232. 10.1663/0007-196X(2007)59%5B226:ANSODM%5D2.0.CO;2

[plb13244-bib-0025] Han Y. , Dai C. , Yang C.F. , Wang Q.F. , Motley T.J. (2008) Anther appendages of *Incarvillea* trigger a pollen‐dispensing mechanism. Annals of Botany, 102, 473–479. 10.1093/aob/mcn102 18567596 PMC2701800

[plb13244-bib-0026] Harder L.D. , Barclay M.R. (1994) The functional significance of poricial anthers and buzz pollination: controlled pollen removal from *Dodecatheon* . Functional Ecology, 8, 509–517. 10.2307/2390076

[plb13244-bib-0027] Harder L.D. , Thomson J.D. (1989) Evolutionary options for maximizing pollen dispersal of animal‐pollinated plants. The American Naturalist, 133, 323–344. 10.1086/284922

[plb13244-bib-0028] Hermann P.M. , Palser B.F. (2000) Stamen development in Ericaceae, I. Anther wall, microsporogenesis, inversion, and appendages. American Journal of Botany, 87, 934–957. 10.2307/2656993 10898771

[plb13244-bib-0029] Hope R.M. (2013) Rmisc: Ryan Miscellaneous. R package version 1.5. Available at: https://CRAN.R‐project.org/package=Rmisc8 (accessed 02 May 2018).

[plb13244-bib-0030] King M.J. , Buchmann S.L. (1996) Sonication dispensing of pollen from *Solanum laciniatum* flowers. Functional Ecology, 10, 449–456. 10.2307/2389937

[plb13244-bib-0031] Köppen W. (1918) Klassifikation der klimate nach temperatur, Niederschlag und Jahreslauf. Petermanns Geographische Mitteilungen, 64, 193–203.

[plb13244-bib-0032] Kuznetsova A. , Brockhoff P.B. , Christensen R.H.B. (2017) lmerTest Package: tests in linear mixed effects models. Journal of Statistical Software, 82, 1–26. Available at: https://cran.r‐project.org/web/packages/lmerTest/lmerTest.pdf (accessed 02 May 2018).

[plb13244-bib-0033] Larson B.M.H. , Barrett S.C.H. (1999) The pollination ecology of buzz‐pollinated *Rhexia virginica* (Melastomataceae). American Journal of Botany, 86, 502–511. 10.1046/j.1365-2745.1999.00362.x 10205070

[plb13244-bib-0034] Luo Z. , Zhang D. , Renner S.S. (2008) Why two kinds of stamens in buzz‐pollinated flowers? Experimental support for Darwin’s division‐of‐labour hypothesis. Functional Ecology, 22, 794–800. 10.1111/j.1365-2435.2008.01444.x

[plb13244-bib-0035] Mendes S.L. , Padovan M.P. (2000) Estação Biológica de Santa Lúcia, Santa Teresa, Espírito Santo. Boletim do Museu de Biologia Mello Leitão, 11(12), 7–34.

[plb13244-bib-0036] Michelangeli F.A. , Guimarães P.J.F. , Penneys D.S. , Almeda F. , Kriebel R. (2013) Phylogenetic relationships and distribution of New World Melastomeae (Melastomataceae). Botanical Journal of the Linnean Society, 171, 38–60. 10.1111/j.1095-8339.2012.01295.x

[plb13244-bib-0037] Michelsen A. , Fink F. , Gogala M. , Traue D. (1982) Plants as transmission channels for insect vibrational songs. Behavioral Ecology and Sociobiology, 11, 269–281. 10.1007/BF00299304

[plb13244-bib-0038] Minnaar C. , Anderson B. , Jager M.L.J. , Karron J.D. (2019) Plant–pollinator interactions along the pathway to paternity. Annals of Botany, 123, 225–245. 10.1093/aob/mcy167 30535041 PMC6344347

[plb13244-bib-0039] Morgan T. , Whitehorn P. , Lye G.C. , Vallejo‐Marín M. (2016) Floral sonication is an innate behaviour in Bumblebees that can be fine‐tuned with experience in manipulating flowers. Journal of Insect Behavior, 29, 233–241. 10.1007/s10905-016-9553-5 27194824 PMC4841848

[plb13244-bib-0040] Muchhala N. (2007) Adaptive trade‐off in corolla shape mediates specialization for flowers pollinated by bats and hummingbirds. The American Naturalist, 169, 494–504. 10.1086/512047 17427121

[plb13244-bib-0015] R Development Core Team . (2012) R: a language and environment for statistical computing. R Foundation for Statistical Computing, Vienna, Austria. Available at: https://www.R‐project.org/ (accessed 02 May 2018).

[plb13244-bib-0041] Renner S.S. (1989) A survey of reproductive biology in Neotropical Melastomataceae and Memecylaceae. Annals of the Missouri Botanical Garden, 76, 496–518. 10.2307/2399497

[plb13244-bib-0042] Renner S.S. (1993) Phylogeny and classification of the Melastomataceae and Memecylaceae. Nordic Journal of Botany, 13, 519–540. 10.1111/j.1756-1051.1993.tb00096.x

[plb13244-bib-0043] Rosi‐Denadai C.A. , Araújo P.C.S. , Campos L.A.O. , Cosme L. Jr , Guedes R.N.C. (2018) Buzz pollination in neotropical bees: genus‐dependent frequencies and lack of optimal frequency for pollen release. Insect Science, 27, 1–10. 10.1111/1744-7917.12602 29740981

[plb13244-bib-0044] Sargent R.D. , Vamosi J.C. (2008) The Influence of Canopy Position, Pollinator Syndrome, and Region on Evolutionary Transitions in Pollinator Guild Size. International Journal of Plant Sciences, 169, 39–47. 10.1086/523359

[plb13244-bib-0045] Solís‐Montero L. , Cáceres‐García S. , Alavez‐Rosas D. , García‐Crisóstomo J.F. , Vega‐Polanco M. , Grajales‐Conesa J. , Cruz‐López L. (2018) Pollinator preferences for floral volatiles emitted by dimorphic anthers of a buzz‐pollinated herb. Journal of Chemical Ecology, 44, 1058–1067. 10.1007/s10886-018-1014-5 30191434

[plb13244-bib-0046] Switzer C.M. , Combes A.S. (2017) Bumblebee sonication behavior changes with plant species and environmental conditions. Apidologie, 48, 223–233. 10.1007/s13592-016-0467-1

[plb13244-bib-0047] Thorp R.W. (2000) The collection of pollen by bees. Plant Systematics and Evolution, 222, 211–223. 10.1007/BF00984103

[plb13244-bib-0048] Vallejo‐Marín M. (2019) Buzz pollination: studying bee vibrations on flowers. New Phytologist, 224, 1068–1074. 10.1111/nph.15666 30585638

[plb13244-bib-0049] Vallejo‐Marín M. , da Silva E.M. , Sargent R.D. , Barret S.C.H. (2010) Trait correlates and functional significance of heteranthery in flowering plants. New Phytologist, 188, 418–425. 10.1111/j.1469-8137.2010.03430.x 20819173

[plb13244-bib-0050] Vallejo‐Marín M. , Manson J.S. , Thomson J.D. , Barret S.C.H. (2009) Division of labor within flowers: heteranthery, a flower strategy to reconcile contrasting pollen fates. Journal of Evolutionary Biology, 22, 828–839. 10.1111/j.1420-9101.2009.01693.x 19320798

[plb13244-bib-0051] Velloso M.S.C. , Brito V.L.G. , Caetano A.P.S. , Romero R. (2018) Anther specializations related to the division of labor in *Microlicia cordata* (Spreng.) Cham. (Melastomataceae). Acta Botanica Brasilica, 32, 349–358. 10.1590/0102-33062017abb0358

[plb13244-bib-0052] Wickham H. (2011) plyr: The Split‐Apply‐Combine Strategy for Data Analysis. Journal of Statistical Software, 40, 1–29. Available at: https://cran.r‐project.org/web/packages/plyr/plyr.pdf (accessed 02 May 2018).

[plb13244-bib-0053] Wickham H. (2016) ggplot2: Elegant Graphics for Data Analysis. Springer, New York, USA. Available at: https://cran.r‐project.org/package=ggplot2/ggplot2.pdf (accessed 02 May 2018).

